# Multi-Response Optimization of Pyrrolizidine Alkaloids Removal from *Chrysanthemum morifolium* by High-Pressure Extraction

**DOI:** 10.3390/foods11233827

**Published:** 2022-11-27

**Authors:** Hao Wang, Qiang Wang, Aiping Lai, Jiahong Zhu, Xiuzhu Huang, Guixian Hu

**Affiliations:** 1 Institute of Agro-product Safety and Nutrition, Zhejiang Academy of Agricultural Sciences, 298 Deshengzhong Road, Hangzhou 310021, China; 2 Institute for the Control of Agrochemicals, Ministry of Agriculture and Rural Affairs, 22 Maizidian Road, Beijing 100125, China

**Keywords:** high-pressure extraction, *Chrysanthemum morifolium*, pyrrolizidine alkaloids, response surface methodology

## Abstract

As an ingredient in various foods, *Chrysanthemum morifolium* flower is popular due to its multiple health benefits. Pyrrolizidine alkaloids (PAs) are hepatotoxic secondary metabolites in *Chrysanthemum* family. Effects of high-pressure extraction (HPE) on PAs removal efficiency, as well as the retention efficiency of functional components, including chlorogenic acid, luteolin-7-β-D-glucopyranoside, 3,5-dicaffeyl quinic acid and total flavonoids, were investigated and optimized using response surface methodology (RSM). Pressure (0.1–200 MPa), numbers of cycles (1–5) and acetic acid concentration (0–10%) were chosen as the independent variables. The results indicated that the pressure was the most significant factors affecting all responses. The optimum HPE for removing Pas and retaining functional components were set at 124 MPa, with one cycle and with an acetic acid concentration of 10%. After comparing the experimental optimum values and predicted optimum values, the validity of RSM model was proved.

## 1. Introduction

*Chrysanthemum* is an edible plant and its flower is often used as an ingredient in a variety of foods, such as soups, porridge and pastries [1], especially in East Asia. There are many varieties of edible *Chrysanthemum*, among which *Chrysanthemum morifolium* has been cultivated for about 370 years in China and is one of the popular cultivars with the best reputation among consumers [2]. As the “Hometown of *Chrysanthemum morifolium* in China”, Tongxiang City has about 3400 hectares of planting area, and its annual output is about 9000 tons and exports about 2000 tons. *Chrysanthemum morifolium* is rich in various functional components that are beneficial to health. Chlorogenic acid and luteolin-7-β-D-glucopyranoside perform biological capacities, such as antioxidant and antiinflammatory functions, as well as hypolipidemic abilities [3]. 3,5-dicaffeyl quinic acid has been reported to inhibit the activity of human immunodeficiency virus-1 (HIV-1) integrase [4]. The Chinese Pharmacopoeia (ChP) has lower limits for the contents of chlorogenic acid (0.20%), luteolin-7-β-D-glucopyranoside (0.08%) and 3,5-dicaffeyl quinic acid (0.70%) in *Chrysanthemum* products. In addition, the content of flavonoids, which have antioxidant, antibacterial, antiinflammatory, antivirus, anticancer and antiaging activities [5], is also an important parameter to evaluate the nutraceutical value of *Chrysanthemum*. Therefore, *Chrysanthemum morifolium* was considered as a food-medicine homologous food and to contribute to cardiovascular protection, antiallergy, antiinflammation, antioxidation and so on [6].

Pyrrolizidine alkaloids (PAs) are secondary metabolites of plants, which are widely distributed in about 3% of the world’s flowering plants. Some types of PAs are capable of suffering N-oxidation to generate their N-oxide forms (PANOs). At present, more than 660 PAs (including their PANOs) have been identified in over 6000 plants. Most of these plants belong to the *Asteraceae*, *Boraginaceae*, *Orchidaceae* and *Fabaceae* families. Han et al. [7] tested 14 PAs/PANOs in 15 *Chrysanthemum*; only jacobine-N-oxide was detected in 60% of *Chrysanthemum.* Kwon et al. [8] analyzed the content of 21 PAs/PANOs in 20 *Chrysanthemum*, but no PAs/PANOs (including jacobine-N-oxide) was detected. The PAs/PANOs in different varieties of *Chrysanthemum* may be different. Many PAs have been reported to be hepatotoxic [9]. Moreover, the toxicity of different PAs/PANOs is different, which is related to its molecular structure. The United States, New Zealand, Australia and other countries, as well as the European Union, World Health Organization (WHO), European Medicines Agency (EMA), European Food Safety Authority (EFSA) and other organizations, have increasingly paid attention to PAs safety issues, and have put forward normative requirements for PAs content in food/agricultural products or consumer intake [10,11]. Therefore, using technical methods to reduce the content of PAs are helpful to enhance the quality and safety of *Chrysanthemum morifolium* products.

For the plants themselves, PAs are thought to act as defense compounds against insects and herbivores [12]. Therefore, the removal technique is more suitable to be applied in harvested *Chrysanthemum morifolium* products. At present, related research papers have mainly focused on PAs extraction technology in plants. Soxhlet extraction is the most frequently applied technique [13], while several papers have discussed novel extraction routines, such as sonication [14], microwave-assisted extraction [15], supercritical fluid extraction [16] and pressurized extraction [13,17,18]. Although Kopp et al. summarized that pressure is an additional parameter affecting PAs efficient extraction, besides solvent, repetitions and other conventional extraction parameters [19], previous studies used a maximum pressure of 10 MPa. Moreover, as these studies focused only on extraction efficiency, they were usually carried out at high temperatures, which can cause damage to heat-sensitive nutrients. High-pressure extraction (HPE) is a novel nonthermal processing technology treating foods at pressures far above atmospheric pressure. Compared to traditional extraction technology, HPE costs less time and solvent to achieve high extraction efficiency. Meanwhile, HPE causes little damage to nutrients in foods. HPE has been used to extraction various target components from foods [20]; however, no reported study has applied HPE to remove PAs from *Chrysanthemum morifolium*.

Therefore, the purpose of this study was to evaluate the effects of HPE conditions on the removal efficiency (RME) of PAs from *Chrysanthemum morifolium*. Meanwhile, the retention efficiency (RTE) of functional components (chlorogenic acid, luteolin-7-β-D-glucopyranoside, 3,5-dicaffeyl quinic acid and total flavonoids) were the constraints when HPE conditions were optimized using response surface methodology (RSM).

## 2. Materials and Methods

### 2.1. Materials

The edible *Chrysanthemum morifolium* flower samples (10.4% moisture content) were obtained from a breeding base in Tongxiang City and were stored in vacuum at 4 °C before the experiment.

### 2.2. Experimental Design

For the optimization of the HPE parameters, a three-level, three-factor Box-Behnken experimental design was applied due to its high efficiency [21]. The independent variables (factors) were pressure (X_1_), number of cycles (X_2_) and acetic acid (AcOH) concentration (X_3_). The range of independent variables were listed in Table 1, which were determined based on previous studies [13,20] and actual production costs [22]. The dependent variables (responses) were the RME of PAs (Y_1_) and the RTE of chlorogenic acid (Y_2_), luteolin-7-β-D-glucopyranoside (Y_3_), 3,5-dicaffeyl quinic acid (Y_4_) and total flavonoids (Y_5_).

A second-order polynomial model (Equation (1)) was used to fit the relationship between factors and responses:

(1) $$Y_{n}=\beta_{n0}+\sum_{i=1}^{3}\beta_{\mathrm{ni}}X_{i}+\sum_{i=1}^{3}\beta_{\mathrm{nii}}X_{i}^{2}+\sum_{i=1}^{2}\sum_{j=i+1}^{3}\beta_{\mathrm{nij}}X_{i}X_{j}$$

where Y_n_ (*n* = 1–5) is the response, β_n0_, β_ni_, β_nii_ and β_nij_ are the coefficients of the intercept, linear, quadratic and interaction terms, respectively, and X_i_ and X_j_ are coded factors.

### 2.3. High-Pressure Extraction

In total, 15 g of *Chrysanthemum morifolium* was vacuum-packed in a polyethylene pouch together with 150 mL of AcOH solution and soaked at 25 °C for 30 s. Afterward, these pouched were pressured in a laboratory-scale high-pressure equipment (UHPF-750, Kefa, Baotou, China) with water as the pressure transfer medium. Once the set value was reached, pressure was released immediately. After cycles of HPE, the samples were drained and dried in an air oven at 30 °C until the average moisture content was close to the original value. All parameters were set as listed in Table 1. All experiments were triplicated.

### 2.4. Determination of PAs via Liquid Chromatographic Separation and Mass Spectrometric Detection (LC–MS/MS)

The PAs contents of all samples were determined via liquid chromatographic separation and mass spectrometric detection (LC–MS/MS) according to Zhang et al. [23] with some modifications. Firstly, the *Chrysanthemum morifolium* samples were ground and sieved through a 60-mesh screen, and then 1× *g* (exact to 0.0001 g) of finely ground *Chrysanthemum morifolium* was extracted with 10 mL of 0.1 M sulfuric acid for 1 min on a vortex mixer (DMT-2500, Jinwen, Shanghai, China) at 1000 rpm, followed by sonication for 15 min. Afterward, the samples were centrifuged (TGL-16B, Anke, Shanghai, China) at 5000× *g*. The supernatant was removed and the residue was extracted again with 10 mL of 0.1 M sulfuric acid as mentioned before. Both supernatants were combined and were subjected to solid phase extraction (SPE) using Cleanert PCX cartridges (200 mg/6 mL, Agela Technologies, Tianjin, China) preconditioned with 5 mL of methanol followed by 5 mL of water. Then the cartridges were washed twice with 5 mL of water followed by 5 mL of 1% (*v/v*) formic acid. Elution was performed with 5 mL of ammonia (0.5% in methanol). The eluate fraction was dried under a gentle stream of nitrogen at 40 °C and redissolved by shaking with 1 mL of methanol/water (10/90, *v/v*). The solutions were filtered through a 0.22 µm filter followed by LC-MS/MS analysis.

Liquid chromatography was performed on a high-performance liquid chromatography (HPLC) system (Nexera X2 LC-30AD, Shimadzu, Tokyo, Japan) equipped with a gradient pump with vacuum degasser, an autosampler and a column oven. A Phenomenex Luna C18 column (150 mm × 2 mm, 3 μm; Phenomenex, Aschaffenburg, Germany) and a Filter Unit (0.22 μm; Agela Technologies, China) were used for chromatography. Mobile phase A was prepared by dissolving 315 mg of ammonium formate and 1 mL of formic acid in 999 mL of water. Mobile phase B was prepared by dissolving 315 mg of ammonium formate and 1 mL of formic acid in 999 mL of methanol. After injection of 10 μL, separation was achieved using a gradient program starting with 90% mobile phase A and 10% mobile phase B for 1 min, changing to 10% mobile phase A within 6 min. This gradient was held constant for 1 min and was then changed to 90% mobile phase A within 0.1 min, which was kept constant again for 1.9 min until the end of the run. The total run time was 10 min at a flow rate of 0.3 mL/min. The column oven was set to 40 °C and the autosampler was cooled to 20 °C. MS analysis was performed in the positive multiple reaction monitoring (MRM) mode on a LCMS-8050 triple quadrupole mass spectrometer (Shimadzu, Japan) equipped with an Electro Spray (ESI) Ionization source. Dwell time was chosen to be 20 ms. The calibration curves obtained in MRM mode were used for quantitation; peak areas were compared with calibration curves generated by three repeated injections of known standards at six concentrations (0.2–20 ng/mL). Thirty-four PAs/PANOs standards were used, including echimidine, echimidine-N-oxide, erucifoline, erucifoline-N-oxide, europine, europine-N-oxide, heliotrine, heliotrine-N-oxide, intermedine, intermedine-N-oxide, jacobine, jacobine-N-oxide, lasiocarpine, lasiocarpine-N-oxide, lycopsamine, lycopsamine-N-oxide, monocrotaline, monocrotaline-N-oxide, retrorsine, retrorsine-N-oxide, senecionine, senecionine-N-oxide, seneciphylline, seneciphylline-N-oxide, senecivernine, senecivernine-N-oxide, senkirkine, trichodesmine, retronecine, indicine, indicine-N-oxide, 7-acetylintermedine, 7-acetyllycopsamine and 7-acetylintermedine N-oxide. The results were expressed in μg/kg of *Chrysanthemum morifolium*.

The PAs’ RME (%) was calculated using Equation (2) below:

(2) $$\mathrm{RME}=\left(1-\frac{C_{1}m_{1}}{C_{0}m_{0}}\right)\times 100\%$$

where $m_{0}$ and $m_{1}$ are the masses of *Chrysanthemum morifolium* before and after the HPE treatment, respectively, and $C_{0}$ (μg/kg) and $C_{1}$ (μg/kg) are the total concentrations of PAs in *Chrysanthemum morifolium* before and after the HPE treatment, respectively.

### 2.5. Determination of Functional Components

#### 2.5.1. Chlorogenic Acid, Luteolin-7-β-D-Glucopyranoside and 3,5-Dicaffeyl Quinic Acid

The chlorogenic acid, luteolin-7-β-D-glucopyranoside and 3,5-dicaffeyl quinic acid content of all samples were determined via HPLC according to Long et al. [2], with some modifications. Powdered sample weighing 0.25 g (exact to 0.0001 g) was extracted with 25 mL of 70% methanol by sonication for 40 min. Afterward, the samples were centrifuged at 5000× *g*. The supernatant was filtered through a 0.22 µm filter followed by HPLC analysis.

HPLC was performed on a Waters 2998 (Waters, Milford, MA, USA) with C18 column (250 mm × 4.6 mm, 5 μm; Shiseido, Tokyo, Japan). Mobile phase A was acetonitrile and mobile phase B was 0.1% phosphoric acid. After injection of 5 μL, separation was achieved using a gradient program starting with 10% mobile phase A and 90% mobile phase B, changing to 18% mobile phase A within 11 min. This gradient was then changed to 20% mobile phase A within 19 min, which was kept constant for 10 min until the end of the run. The total run time was 40 min at a flow rate of 1 mL/min. The column temperature was maintained at 35 °C. The detection wavelength was 348 nm.

The chlorogenic acid, luteolin-7-β-D-glucopyranoside and 3,5-dicaffeyl quinic acid concentrations of supernatant were calculated by comparing the peak areas of samples with the peak area of standards (containing 35 mg/L of chlorogenic acid, 25 mg/L of luteolin-7-β-D-glucopyranoside and 80 mg/L of 3,5-dicaffeyl quinic acid in 70% methanol). The results were expressed in g/100 g of *Chrysanthemum morifolium*.

#### 2.5.2. Total Flavonoids

The total flavonoids content of all samples was determined according to Lai et al. [24], with some modifications. Powdered sample weighing 0.1 g (exact to 0.0001 g) was mixed with 25 mL of methanol and shaken (160 rpm) at 65 °C for 2 h. Afterward, the samples were centrifuged at 5000× *g*. One mL of the supernatant was mixed with 2 mL of AlCl_3_ (0.1 M) and 3 mL of potassium acetate (1 M), and then was diluted to 10 mL with 70% methanol. After 30 min of reaction, the absorbance of the solution at 420 nm was measured with spectrophotometer (UV-6100, Yuanxi, Shanghai, China). For calibration curves, 1 mL of rutin standard solution (0.01–0.15 mg/mL) was used instead of 1 mL of supernatant. The results were expressed in g/100 g of *Chrysanthemum morifolium*.

#### 2.5.3. Retention Efficiency (RTE)

The RTE (%) of functional components were calculated using Equation (3) below:

(3) $$\mathrm{RTE}=\frac{C_{1}m_{1}}{C_{0}m_{0}}\times 100\%$$

where $m_{0}$ and $m_{1}$ are the masses of *Chrysanthemum morifolium* before and after the HPE treatment, respectively, and $C_{0}$ (%) and $C_{1}$ (%) are the concentrations of chlorogenic acid, luteolin-7-β-D-glucopyranoside, 3,5-dicaffeyl quinic acid and total flavonoids in *Chrysanthemum morifolium* before and after the HPE treatment, respectively.

### 2.6. Statistical Analysis

The second-order polynomial models in this study were fitted using the experimental data and were analyzed with Design-Expert software (version 12.0.3.0, Stat-Ease, Inc., Minneapolis, MN, USA). The initial models were further improved by reducing insignificant terms. The efficiency of the final models was investigated by determining the *p*-value of the regression equation, the number of significant terms, the *p*-value of the lack of fit test and the coefficients of determination (R^2^) and adjusted R^2^ [20]. For the good fit of a model, the R^2^ value should be at least 0.80 [25]. The closer R^2^ to 1 and the closer adjusted R^2^ is to R^2^, the closer the fitting model is to the experimental value. The lack of fit test was used to judge the acceptability of regression model. A satisfied regression model requires an insignificant lack of fit. The Fisher F-test was used to compare the significance of each term in the final model. Three-dimensional (3D) response surface plots were to see graphically how the responses change with respect to the factors of significant (*p* < 0.05) interactions. The numerical optimization procedure was applied to predict the optimum level of factors for the highest PAs’ RME and functional components RTE.

## 3. Results and Discussion

### 3.1. PAs’ Removal Efficiency

**Figure 1:**
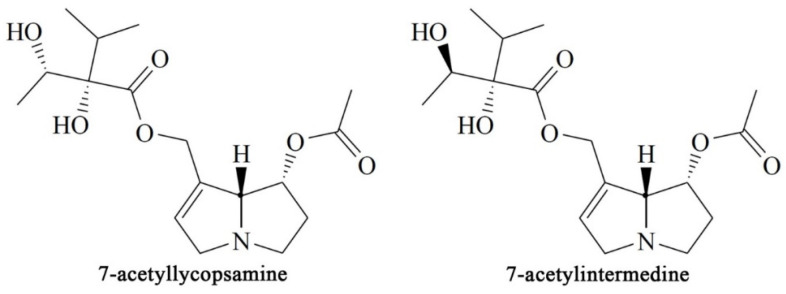
Structures of PAs detected in *Chrysanthemum morifolium*.

Two PAs (7-acetylintermedine and 7-acetyllycopsamine, Figure 1) were detected in *Chrysanthemum morifolium*. PA-mediated toxicity is determined by 1,2-unsaturation in the pyrrolizidine ring and an ester function on the side chain. In general, PAs with a 1,2 double bond are considered toxic. Besides, PAs that exert the highest toxicity are cyclic diesters, with monoesters being the ones that cause the lowest level of injuriousness; between them are the open-chain diesters, which cause an intermediary toxicity [26]. Therefore, the detected PAs in *Chrysanthemum morifolium* are potentially toxic and it is necessary to reduce the content.

Table 1 and Table 2 listed the experimental value and the analysis results of the PAs’ RME model, respectively. The quadratic terms of cycles and AcOH concentrations and the interaction terms of pressure and cycles were reduced in the improved model of PAs’ RME, due to insignificance (*p* > 0.05, data not shown). The *p*-value of the regression equation was less than 0.0001, and the R^2^ and adjusted R^2^ were 0.9804 and 0.9686, respectively. The value of the lack of fit was insignificant (*p* = 0.1190 > 0.05). All evaluation parameters indicated the good predictability of the final model.

**Figure 2:**
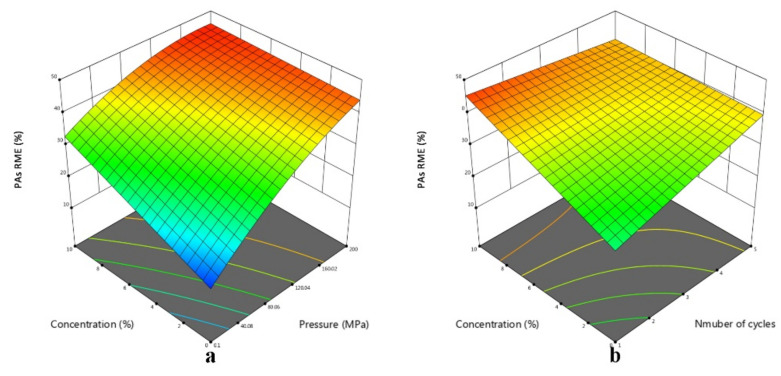
3D surface plots of PAs’ RME: (**a**) pressure–concentration; (**b**) number of cycles–concentration.

Within the experimental values of independent variables, the highest RME (47%) of PAs from *Chrysanthemum morifolium* was achieved after five cycles of HPE at 200 MPa with 5% AcOH solution as solvent (shown in Table 1). Table 3 summarized the F-ratio of each term in the final model of the PAs’ RME. The larger the F-ratio, the more significant the effect of corresponding term on the response [27]. Pressure with an F-ratio of 330.64 was the most significant term positively affecting the PAs’ RME from *Chrysanthemum morifolium*. Consistent with the conclusion summarized by Kopp et al. [19], pressure played a nonnegligible role on removing PAs. They considered that high pressure could increase the boiling point of the solvent, thus setting higher extraction temperature. Differently in this study, even higher pressure was used to achieve nonthermal extraction. The high pressure could improve the permeability of the solvent into the samples and accelerate the transfer of target components into the solvent [28]. Besides, the quadratic term of pressure was negatively correlated with response (β_11_ = −3.68), indicating that the increase in RME induced by pressure was more obvious in the lower pressure range. Similar result was reported by Luo et al. [29] for removing cations with acidic solvent. Following pressure, AcOH concentration had a significant effect on PAs’ RME with 78.00 of F-ratio. Kopp et al. [13] also found that 5% acid solutions (phosphoric acid, formic acid and sulfuric acid, respectively) showed better extraction yield of PAs from *Symphytum officinale* than 1% corresponding acid solutions. It could be attributed to the neutralization reaction between alkaloids and AcOH. Moreover, a positive correlation between cycles of HPE and PAs’ RME was also observed. This result could be explained by continuous enhancement of permeability caused by repeated instantaneous decompression. The release of pressure inside the samples, compared to the external environment, had a short lag, so that the structure of outer cells was destroyed under a huge pressure difference [30]. The interaction items of AcOH concentration with pressure ($X_{1}X_{3}$) and cycles ($X_{2}X_{3}$) were significant, so the 3D response surface plots were generated as shown in Figure 2. According to Figure 2a, with the pressure increasing, the positive effect of AcOH concentration on PAs’ RME was attenuated. Considering that 7-acetylintermedine and 7-acetyllycopsamine are soluble in water [31], this phenomenon indicated that the physical effect of pressure (providing powerful force for the permeation of solvent) is dominant at higher pressure levels, and AcOH concentration only affects the form (ion or molecule) of PAs present in the solvent. Similarly, Figure 2b showed that the increase in the PAs’ RME was more sensitive to repeated HPE when AcOH concentration was at a lower level. In conclusion, HPE conditions influenced the PAs’ RME, and pressure > AcOH concentration > cycles with respect to the order of significance.

### 3.2. Functional Components Retention Efficiency

For chlorogenic acid, we found that all terms were significant (*p* < 0.05) in the initial model of RTE and, therefore, we retained them. For luteolin-7-β-D-glucopyranoside, 3,5-dicaffeyl quinic acid and total flavonoids, the models were improved by reducing insignificant items. Table 2 summarized the results of efficiency analysis of four final RTE models. The fitting degree was satisfied enough to describe the correlation between the predicted and actual values due to the R^2^ (>0.8) of all models. The *p*-value of regression (<0.05) and lack of fit (>0.05) indicated a good relationship between responses and factors.

Pressure had a negative effect on RTE of chlorogenic acid, luteolin-7-β-D-glucopyranoside and 3,5-dicaffeyl quinic acid with the highest F-ratio. For total flavonoids, the quadratic term of pressure had the highest F-ratio. Pu et al. [32], who optimized the HPE conditions of chlorogenic acid from *Honeysuckle* by RSM, also found that the pressure was the most significant factor among HPE conditions. Liu et al. [33] pointed out that pressure over 100 MPa could rupture the florets tissues and cellulose, enhancing the mass transfer of the solvents into the materials and the soluble constituents into the solvents. Besides, they also observed no difference in flavonoids extraction yield from safflower between one cycle and three cycles of HPE. Similarly in the present study, cycles showed insignificant effect on RTE of total flavonoids and luteolin-7-β-D-glucopyranoside (*p* > 0.05). However, HPE cycles was significantly correlated with the RTE of chlorogenic acid and 3,5-dicaffeyl quinic acid (same as PAs). This phenomenon might be due to the different distribution of various components in *Chrysanthemum morifolium*. The β_3_ in four models (Y_2_ to Y_5_) were all greater than zero, indicating that the higher the AcOH concentration, the higher the RTE of four functional components. This is because chlorogenic acid, luteolin-7-β-D-glucopyranoside and 3,5-dicaffeyl quinic acid are acidic, similar to AcOH. Therefore, it could be further inferred that the flavonoids in *Chrysanthemum morifolium* were also acidic. In *Chrysanthemum indicum* flower, ten flavonoids were identified, all of which were slightly acidic [34].

**Figure 3:**
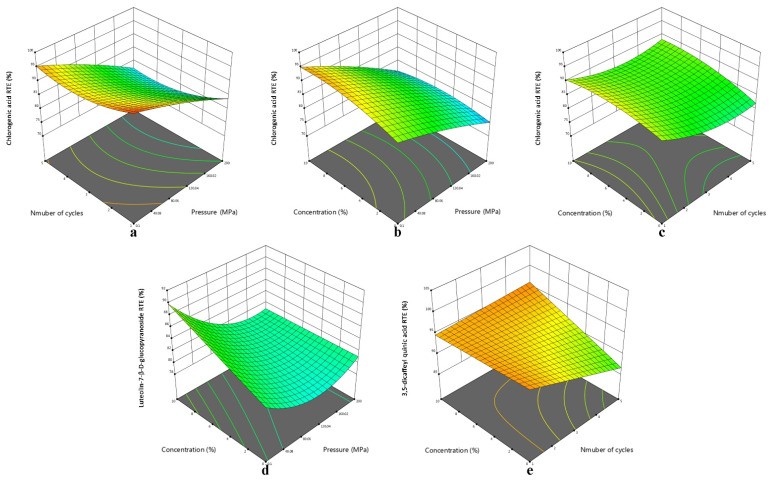
Three-dimensional surface plots of functional components RTE: (**a**) pressure–number of cycles on chlorogenic acid; (**b**) pressure–concentration on chlorogenic acid; (**c**) number of cycles–concentration on chlorogenic acid; (**d**) pressure–concentration on luteolin-7-β-D-glucopyranoside; (**e**) number of cycles–concentration on 3,5-dicaffeyl quinic acid.

In order to show the significant interaction terms more directly, 3D response surface plots were listed in Figure 3. For chlorogenic acid, the interaction of AcOH concentration and cycles showed a positive effect on the response (Figure 3c), while showing a negative effect of two other interaction terms that involved pressure (Figure 3a,b). Overall, lower pressure and fewer cycles were more critical conditions for improved RTE. For luteolin-7-β-D-glucopyranoside, Figure 3d demonstrated that the lower the pressure, the more obvious the effect of the AcOH concentration on the RTE, similar to Figure 2a and Figure 3b. For 3,5-dicaffeyl quinic acid, the interaction term of cycles and AcOH concentration was significantly and positively correlated with the RTE. It could be found in Figure 3e that a higher concentration of AcOH was more conductive to retain 3,5-dicaffeyl quinic acid with multiple cycles of HPE.

### 3.3. Optimization of HPE Conditions and Validation of Predictive Models

Our target was to obtain simultaneously high PAs’ RME and high functional component RTEs, so HPE conditions were optimized using Design-Expert software with some qualifications. The three independent variables were limited in the set range of this study. The lower limit and upper limit were 0% and 100%, respectively, for all five dependent variables. Considering that removal of PAs was the main goal of this study, the importance of PAs’ RME was set as level 5 (highest), while others as default level 3 (moderate). The optimum HPE conditions after initial calculation was 123.794 MPa, with one cycle and an AcOH concentration of 10%. Considering the actual operation of high-pressure equipment, the pressure was simplified to 124 MPa. Based on the simplified HPE conditions, the simplified predicted values of five dependent variables were calculated and were found to be close to the initially predicted values (Table 4). Three independent experiments at simplified optimum conditions were performed to validate the predictive models. The experimental value of PAs’ RME was 47%, and the RTE experimental values of chlorogenic acid, luteolin-7-β-D-glucopyranoside, 3,5-dicaffeyl quinic acid and total flavonoids were 88%, 82%, 90% and 79%, respectively. The closeness between the experimental optimum response and predicted optimum values proved the validity of the recommended optimum extraction condition. In addition, the PAs’ RME under optimum conditions was very close to that of run 17 (as shown in Table 1). Differenly compared with run 17, the RTE of chlorogenic acid and 3,5-dicaffeyl quinic acid increased by 13.6% and 1.3%, respectively, while RTE of luteolin-7-β-D-glucopyranoside and total flavonoids decreased by 2.8% and 5.8%, respectively. Although the extraction efficiency of PAs in this study was not as good as previous related studies, they paid more attention to the recovery of PAs for subsequent purification and/or analysis, so that plants were ground into powder in order to improve efficiency [19]. Differently, the target of this study was to improve the safety and value of whole *Chrysanthemum morifolium* products, so the advantage of this study was that the integrity of the *Chrysanthemum morifolium* product was maintained. In order to reduce product loss, future studies could explore the application of noninvasive and nondestructive testing technologies in the detection of PAs in *Chrysanthemum morifolium* products, such as computer vision-based techniques [35]. Moreover, high-pressure processing was considered to inhibit various metabolic activities in food during storage [36], and therefore, the following work could also focus on the generation and accumulation of PAs in HPE-treated *Chrysanthemum morifolium* during storage.

## 4. Conclusions

The present study applied HPE to removal PAs from *Chrysanthemum morifolium*. Two PAs (7-acetylintermedine and 7-acetyllycopsamine) were detected. The results showed that HPE conditions significantly affected the PAs’ RME and functional components’ (chlorogenic acid, luteolin-7-β-D-glucopyranoside, 3,5-dicaffeyl quinic acid and total flavonoids) RTE, especially pressure. A higher pressure, more cycles and a higher AcOH concentration were desirable to removal of PAs, while higher RTE of functional components required lower pressure, fewer cycles and higher AcOH concentration. The reduced quadratic models were satisfied to describe the relationship between the factors and responses. HPE at 124 MPa, with 1 cycle and an AcOH concentration of 10%, were considered as optimum conditions to achieve higher RME of PAs (47%) as well as higher RTE of chlorogenic acid (88%), luteolin-7-β-D-glucopyranoside (82%), 3,5-dicaffeyl quinic acid (90%) and total flavonoids (79%). Thus, HPE could potentially reduce the PAs risk of *Chrysanthemum morifolium* products.

## Figures and Tables

**Table 1:** Experimental design matrix of HPE conditions and results for PAs’ RME and functional components’ RTE.

Runs	Coded Factors			Uncoded Factors			RME	RTM			
	X_1_	X_2_	X_3_	Pressure (MPa)	Number of Cycles	AcOH Concentration (%)	PAs	Chlorogenic Acid	Luteolin-7-β-D-Glucopyranoside	3,5-Dicaffeyl Quinic Acid	Total Flavonoids
1	−1	−1	0	0.1	1	5	25 ± 0	99 ± 0	90 ± 0	100 ± 1	92 ± 3
2	−1	0	−1	0.1	3	0	13 ± 4	89 ± 2	81 ± 2	95 ± 2	83 ± 3
3	−1	0	1	0.1	3	10	31 ± 1	95 ± 2	90 ± 3	100 ± 4	90 ± 0
4	−1	1	0	0.1	5	5	26 ± 1	95 ± 3	90 ± 1	96 ± 2	90 ± 1
5	0	−1	−1	100.05	1	0	26 ± 1	90 ± 1	81 ± 2	95 ± 1	75 ± 3
6	0	−1	1	100.05	1	10	45 ± 1	90 ± 0	82 ± 1	94 ± 0	79 ± 3
7	0	0	0	100.05	3	5	37 ± 1	86 ± 2	80 ± 1	92 ± 2	81 ± 3
8	0	0	0	100.05	3	5	39 ± 1	87 ± 1	82 ± 1	94 ± 2	79 ± 3
9	0	0	0	100.05	3	5	38 ± 1	86 ± 3	80 ± 3	96 ± 2	79 ± 3
10	0	0	0	100.05	3	5	37 ± 2	87 ± 1	80 ± 1	92 ± 1	78 ± 4
11	0	0	0	100.05	3	5	39 ± 1	87 ± 0	84 ± 3	93 ± 2	78 ± 1
12	0	1	−1	100.05	5	0	41 ± 5	83 ± 2	82 ± 1	86 ± 1	78 ± 2
13	0	1	1	100.05	5	10	42 ± 1	88 ± 1	85 ± 0	96 ± 1	86 ± 1
14	1	−1	0	200	1	5	42 ± 1	84 ± 1	83 ± 1	95 ± 0	86 ± 2
15	1	0	−1	200	3	0	45 ± 1	75 ± 2	80 ± 3	85 ± 3	82 ± 3
16	1	0	1	200	3	10	46 ± 2	76 ± 1	82 ± 1	87 ± 0	84 ± 4
17	1	1	0	200	5	5	47 ± 3	77 ± 1	84 ± 1	89 ± 1	83 ± 3

Values are means ± standard deviations (*n* = 3).

**Table 2:** Results of regression coefficients and final model efficiency analysis.

Source		RME	RTE			
		PAs	Chlorogenic Acid	Luteolin-7-β-D-Glucopyranoside	3,5-Dicaffeyl Quinic Acid	Total Flavonoids
Regression coefficient	β_0_	38.02	86.67	80.54	93.31	79.03
	Linear					
	β_1_	10.72	−8.20	−2.64	−4.42	−2.40
	β_2_	2.36	−2.48	0.50	−1.95	-
	β_3_	5.20	1.70	1.74	2.07	2.60
	Quadratic					
	β_11_	−3.68	−0.91	3.11	-	7.33
	β_22_	-	3.04	2.40	-	-
	β_33_	-	−01.89	-	-	-
	Interaction					
	β_12_	-	−0.86	-	-	-
	β_13_	−4.19	−1.22	−1.82	-	-
	β_23_	−4.48	1.33	-	2.64	-
Lack of fit		0.1190	0.7051	0.4703	0.2860	0.1036
R^2^		0.9804	0.9956	0.8389	0.8357	0.8326
Adjusted R^2^		0.9686	0.9900	0.7423	0.7809	0.7940
*p*-Value (regression)		<0.0001	<0.0001	0.0017	0.0001	<0.0001

**Table 3:** F-ratio of terms in final models.

Terms	RME	RTE			
	PAs	Chlorogenic Acid	Luteolin-7-β-D-Glucopyranoside	3,5-Dicaffeyl Quinic Acid	Total Flavonoids
Linear					
$X_{1}$	330.64	1262.12	17.71	38.32	9.07
$X_{2}$	15.99	115.40	0.63	7.49	-
$X_{3}$	78.00	54.03	7.68	8.38	10.65
Quadratic					
$X_{1}^{2}$	20.63	8.18	12.98	-	44.95
$X_{2}^{2}$	-	90.97	7.71	-	-
$X_{3}^{2}$	-	35.34	-	-	-
Interaction					
$X_{1}X_{2}$	-	6.89	-	-	-
$X_{1}X_{3}$	25.29	13.95	4.20	-	-
$X_{2}X_{3}$	28.87	16.48	-	6.82	-

**Table 4:** Predicted and experimental values of dependent variables at optimum conditions.

Terms	RME (%)	RTE (%)			
	PAs	Chlorogenic Acid	Luteolin-7-β-D-Glucopyranoside	3,5-Dicaffeyl Quinic Acid	Total Flavonoids
Initial predicted values	47	89	83	94	81
Simplified predicted values	47	89	83	94	81
Experimental values	47 ± 0	88 ± 1	82 ± 1	90 ± 1	79 ± 2

Experimental values are means ± standard deviations (*n* = 3).

## Data Availability

The data presented in this study are available on request from the corresponding author.
